# Supplementary material for: A rapid and robust method for single cell chromatin accessibility profiling
Source: Nat Commun. 2018 Dec 17;9:5345. doi: 10.1038/s41467-018-07771-0 (PMC6297232; doi:10.1038/s41467-018-07771-0)
Supplement: Supplementary file 1 — Supplementary Infomation [file 41467_2018_7771_MOESM1_ESM.pdf]

A rapid and robust method for single cell chromatin accessibility profiling

Chen *et al.*

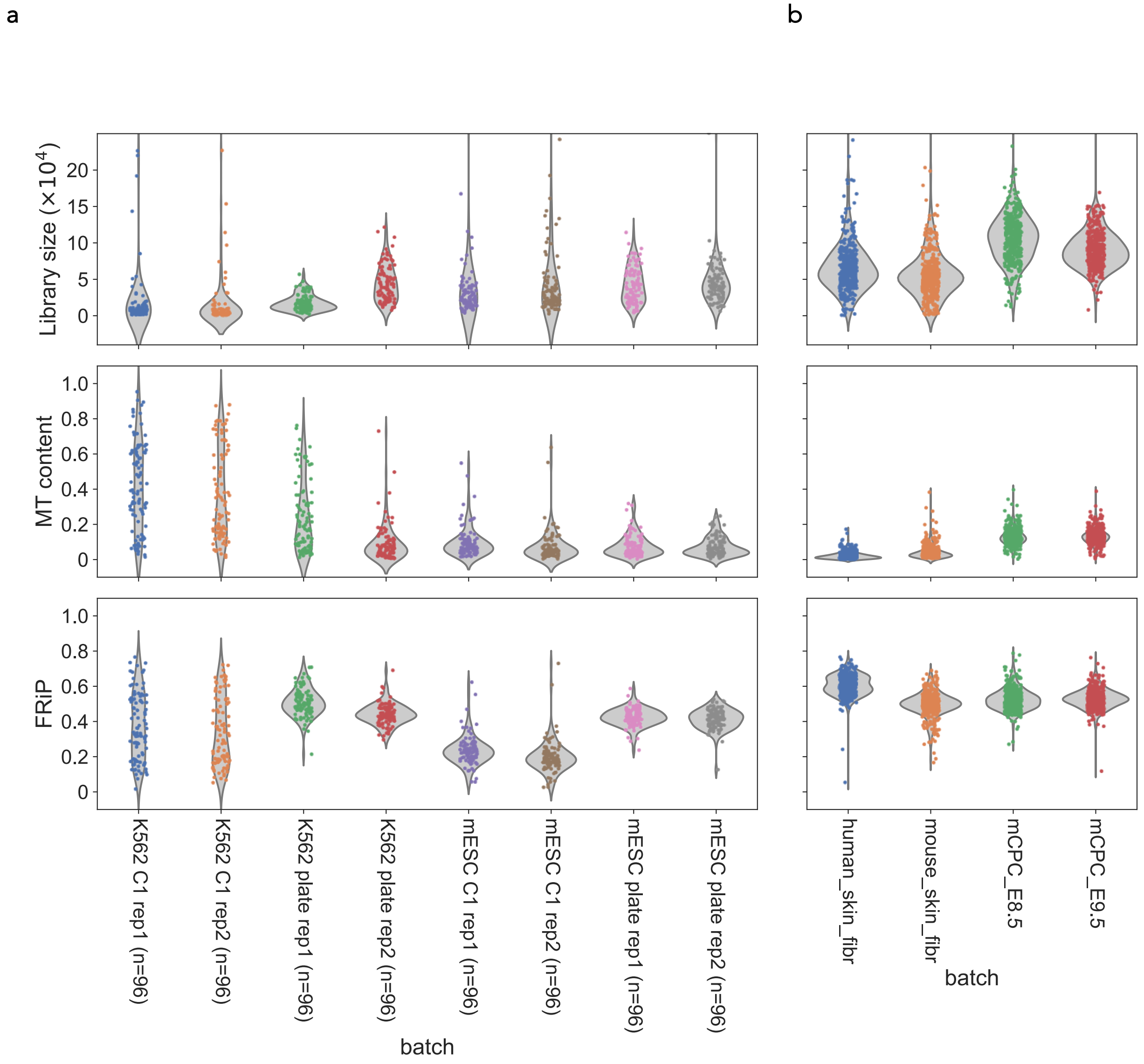

Supplementary Figure 1. (a) Violin plots showing the comparisons of distributions of the median library size (estimated by the Picard tool), fraction of mitochondrial DNA (MT content) and fraction of reads in peaks (FRiP) in single cells from either plate or C1 scATAC-seq approach. **(b)** the same metrics as in **(a)** showing data obtained from cryopreserved cells of four different primary tissues.

a

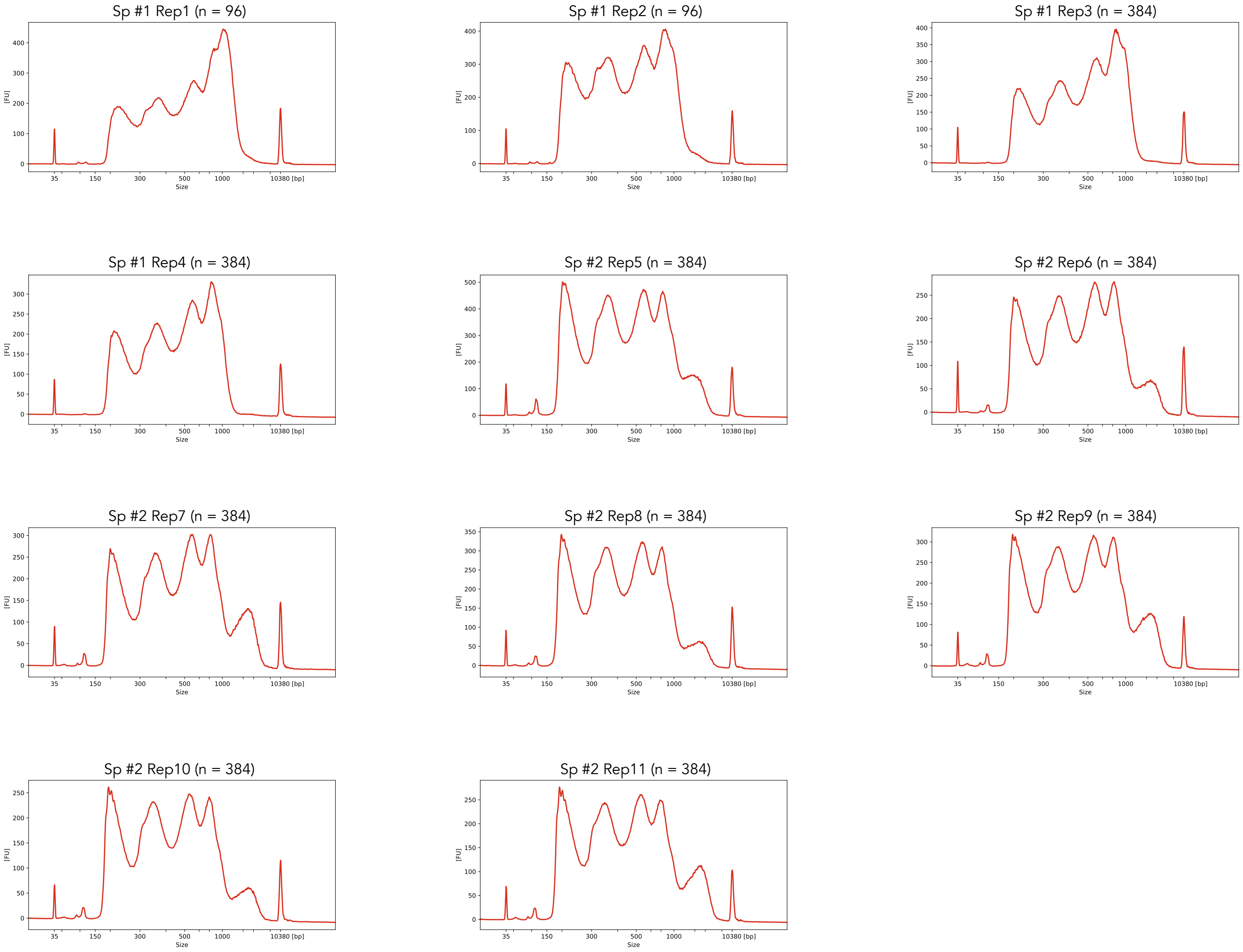

b

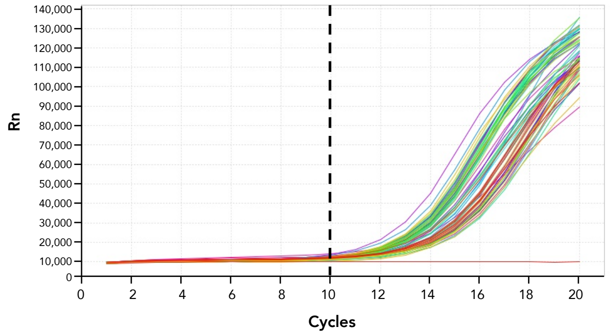

Supplementary Figure 2. (**a**) Bioanalyzer results of pools of 11 different plates (two spleens) of scATAC-seq in this study. (**b**) qPCR amplification plot of 64 different single cell libraries. The qPCR was performed after 8 cycles of pre-amplification. Dotted line indicates the number of cycles used for final amplification. A total of 8 + 10 = 18 cycles were performed in this study.

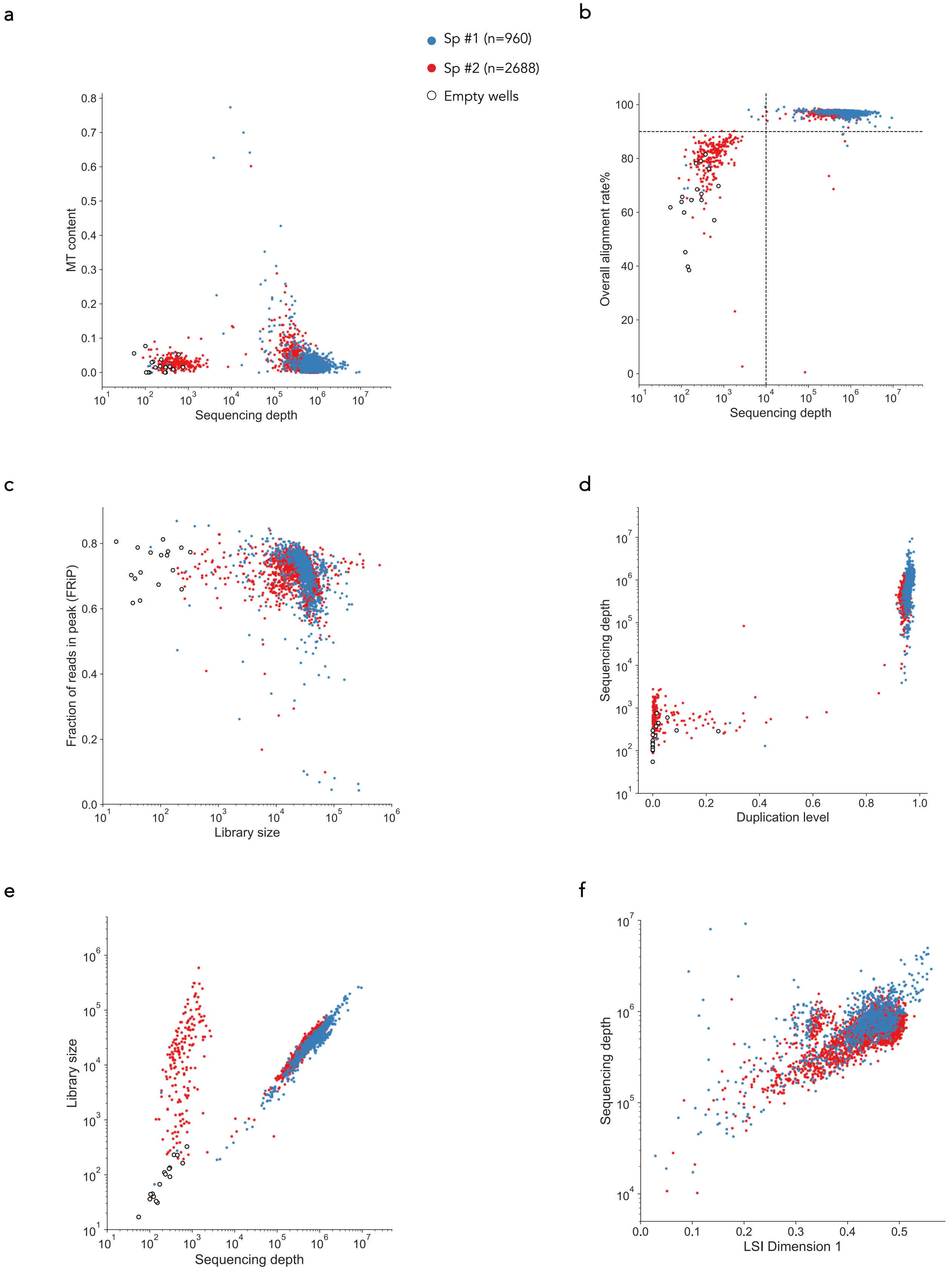

Supplementary Figure 3. Scatter plots of different quality control metrics. Single cells from different batches are indicated by different colours, and empty well controls are also indicated. We removed cells that have less than 10,000 reads or less than 90% mapping rate, as indicated by dotted lines in (b). Sp: spleen; MT content: fraction of mitochondrial reads; LSI: latent semantic indexing; Library size is estimated by the Picard tool.

a

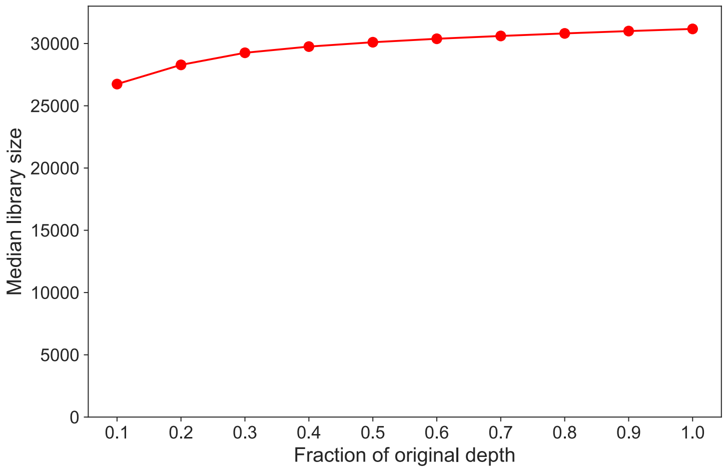

b

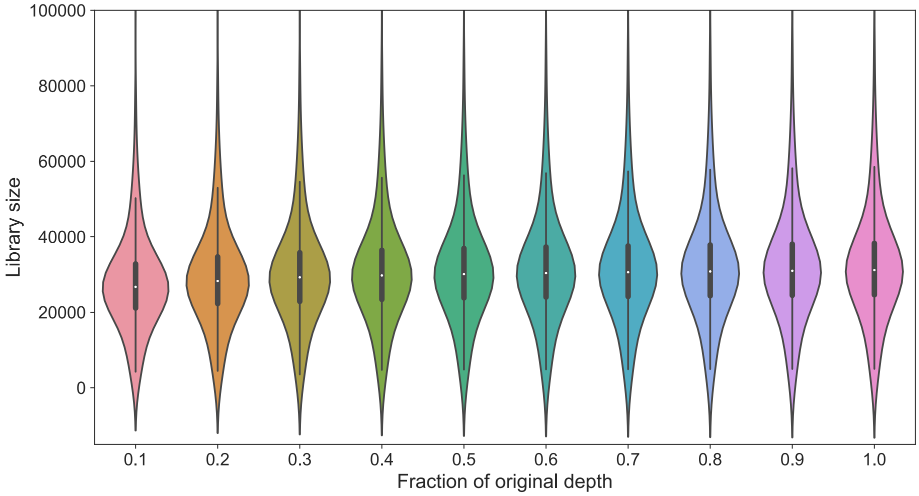

Supplementary Figure 4. (**a**) The median library size after downsampling (at the fastq stage) to different fractions relative to the full data sets. (**b**) Violin Plot of the library size at the different level of downsampling.

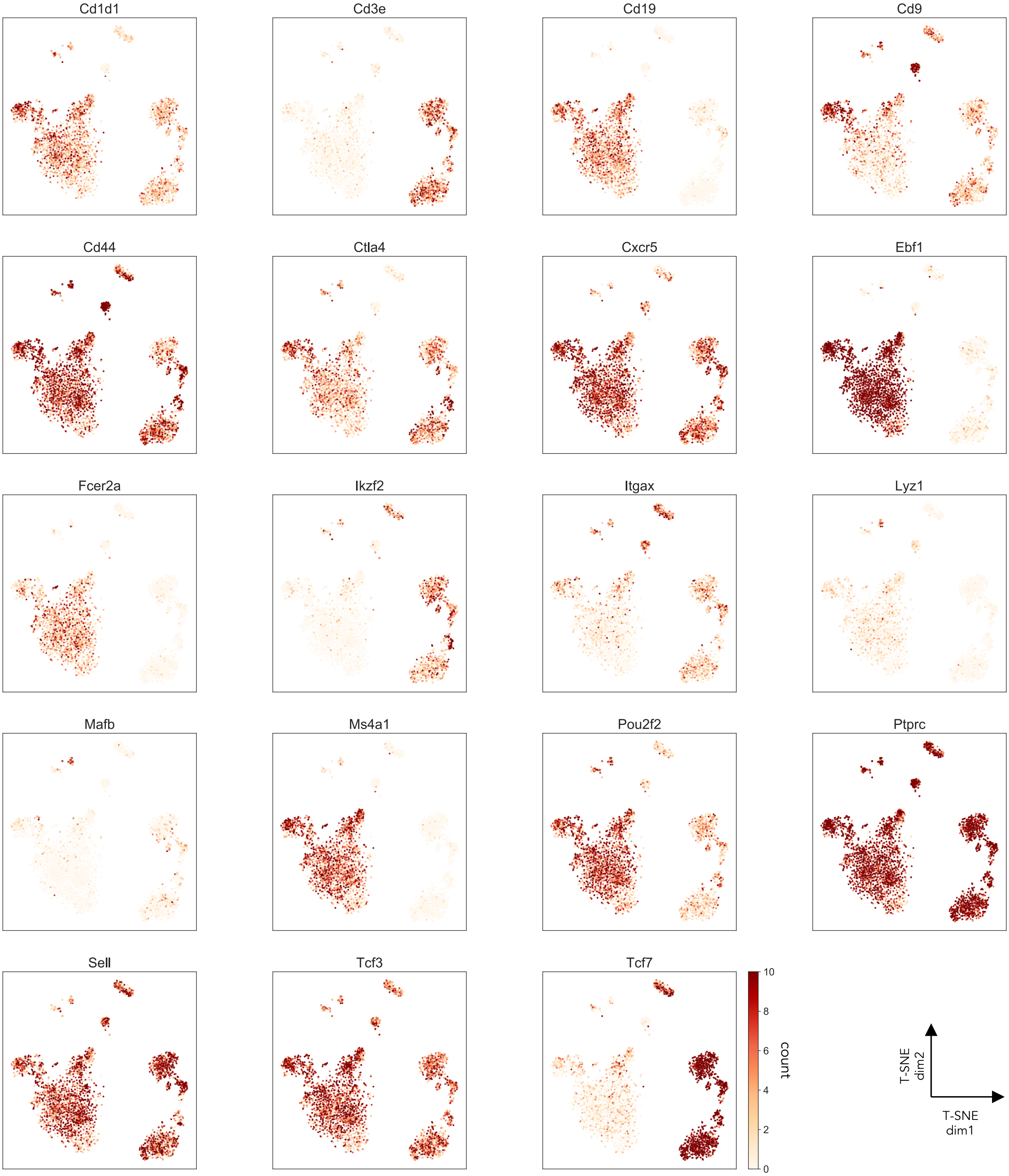

Supplementary Figure 5. Number of counts from all peaks that assigned to the indicated genes by HOMER.

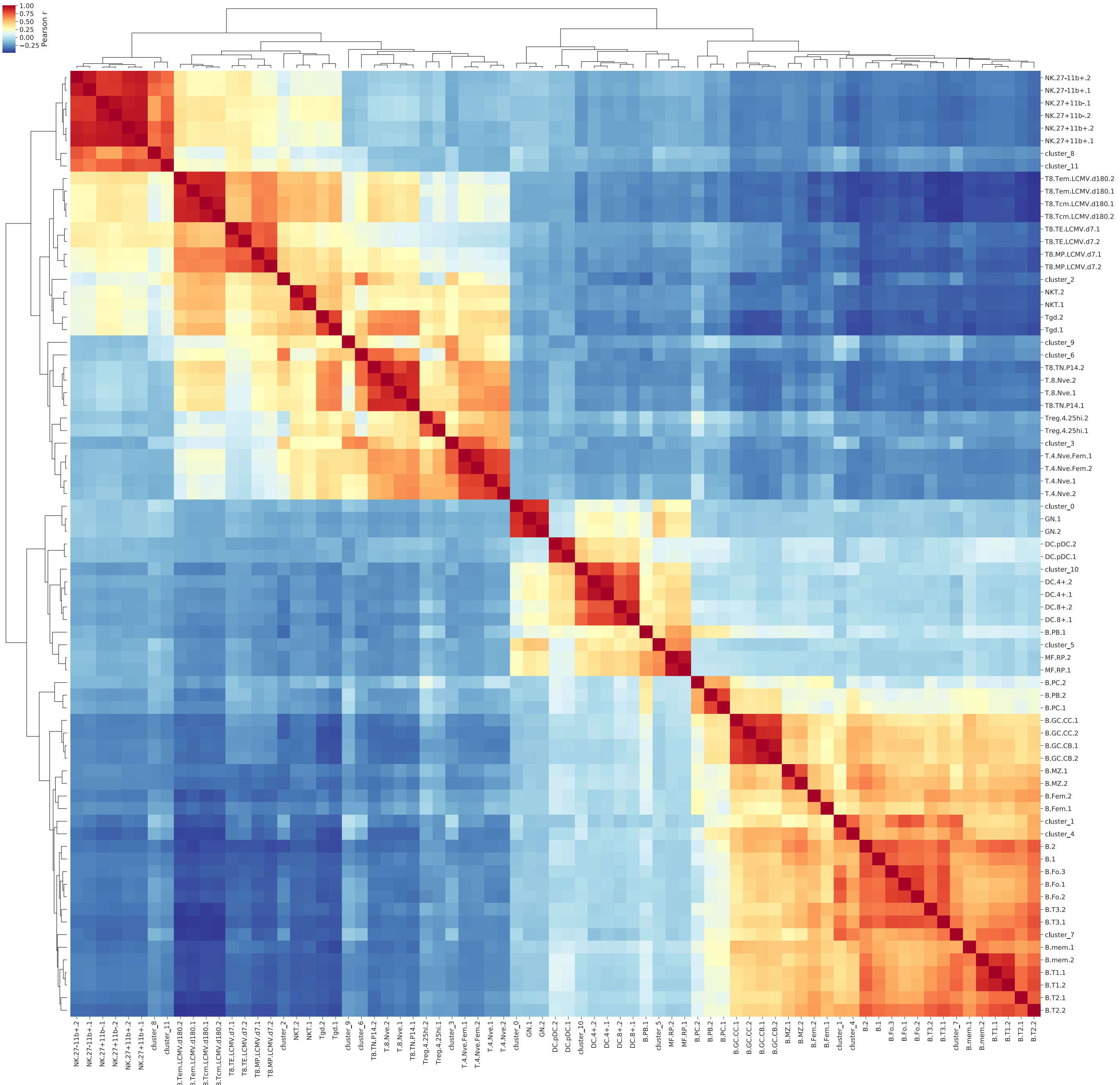

Supplementary Figure 6. Hierarchical clustering of the Pearson's correlation between aggregated single cell clusters and the bulk ATAC-seq data sets from ImmGen. The full matrix is shown here, and the ImmGen sample labels were taken directly from the ImmGen ATAC-seq data deposited at the European Nucleotide Archive (ENA) (https://www.ebi.ac.uk/ena/data/view/PRJNA392905).

cluster_0

```
GN.1       0.858115
GN.2       0.845666
MF.RP.1    0.255730
MF.RP.2    0.244122
DC.4+.1    0.204005
Name: cluster_0, dtype: float64
```

cluster_1

```
B.Fo.1    0.774211
B.Fo.2    0.735329
B.T3.1    0.678556
B.Fo.3    0.672733
B.T3.2    0.575539
Name: cluster_1, dtype: float64
```

cluster_2

```
T8.Tcm.LCMV.d180.1    0.554817
T8.Tcm.LCMV.d180.2    0.538327
T8.Tem.LCMV.d180.1    0.516215
Tgd.1                 0.491312
T8.Tem.LCMV.d180.2    0.478378
Name: cluster_2, dtype: float64
```

cluster_3

```
T.4.Nve.Fem.2    0.750628
T.4.Nve.Fem.1    0.748461
T.4.Nve.1        0.592180
T.4.Nve.2        0.547945
T8.TN.P14.2      0.494832
Name: cluster_3, dtype: float64
```

cluster_4

```
B.MZ.2    0.606645
B.Fo.1    0.592016
B.Fo.2    0.567450
B.2       0.565281
B.MZ.1    0.560363
Name: cluster_4, dtype: float64
```

cluster_5

```
MF.RP.1    0.635129
MF.RP.2    0.616450
GN.1       0.485931
GN.2       0.466141
B.PB.1     0.397456
Name: cluster_5, dtype: float64
```

cluster_6

```
T.8.Nve.2      0.711286
T8.TN.P14.2    0.710398
T8.TN.P14.1    0.563161
T.8.Nve.1      0.552890
Tgd.1          0.477801
Name: cluster_6, dtype: float64
```

cluster_7

```
B.T2.2      0.730364
B.T1.2      0.723927
B.T1.1      0.697820
B.mem.2     0.697162
B.T3.1      0.681272
Name: cluster_7, dtype: float64
```

cluster_8

```
NK.27-11b+.2    0.764921
NK.27-11b+.1    0.702963
NK.27+11b+.2    0.698239
NK.27+11b+.1    0.684591
NK.27+11b-.2    0.568392
Name: cluster_8, dtype: float64
```

cluster_9

```
Treg.4.25hi.1    0.455224
T.4.Nve.Fem.2    0.399113
T.4.Nve.Fem.1    0.390887
Treg.4.25hi.2    0.357982
NKT.2            0.347487
Name: cluster_9, dtype: float64
```

cluster_10

```
DC.4+.1     0.829736
DC.4+.2     0.796127
DC.8+.1     0.725133
DC.8+.2     0.654761
DC.pDC.1    0.470827
Name: cluster_10, dtype: float64
```

cluster_11

```
NK.27+11b+.2    0.793500
NK.27+11b+.1    0.755976
NK.27+11b-.1    0.721280
NK.27+11b-.2    0.720818
NK.27-11b+.2    0.706145
Name: cluster_11, dtype: float64
```

Supplementary Figure 7. The top correlated ImmGen bulk samples to each aggregated single cell clusters. Top 5 pearson r scores for each cluster are shown.

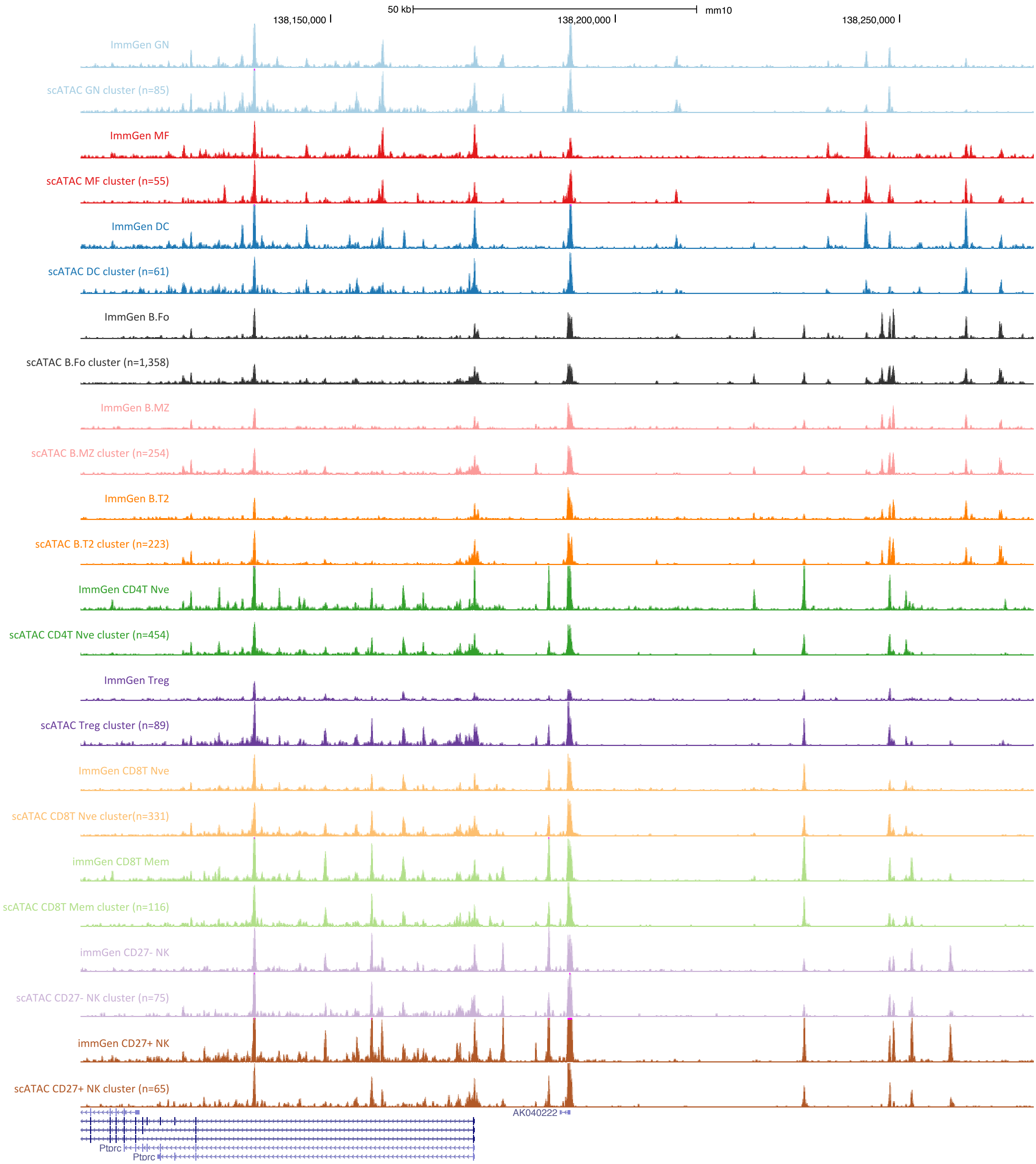

Supplementary Figure 8. UCSC genome browser tracks showing ATAC-seq profiles of indicated ImmGen bulk samples and aggregated single cell clusters around the *Ptprc* (*Cd45*) promoter region.

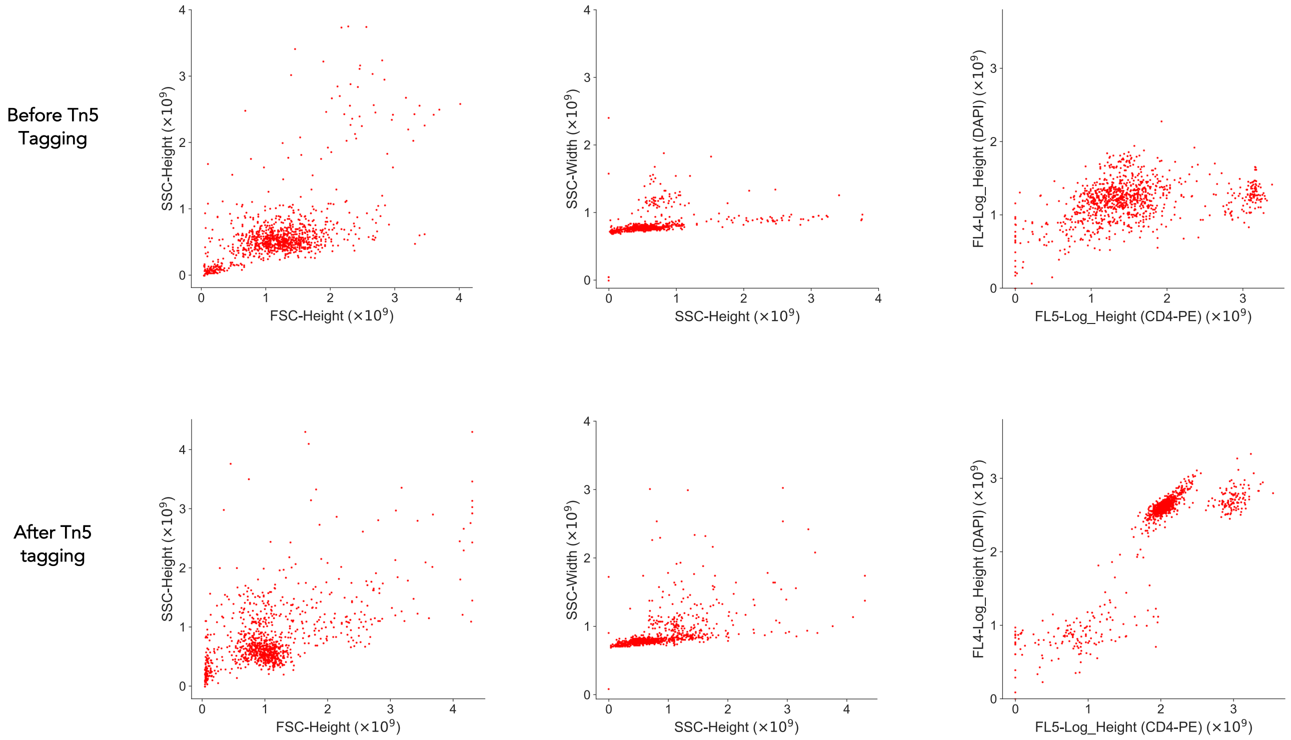

Supplementary Figure 9. FACS results showing the anti-CD4-PE and DAPI stain on mouse splenocytes before (top) and after (bottom) Tn5 tagging. Note, all cells are DAPI negative before Tn5 tagging but become DAPI positive afterwards. CD4-PE signal remains after Tn5 tagging.

a b

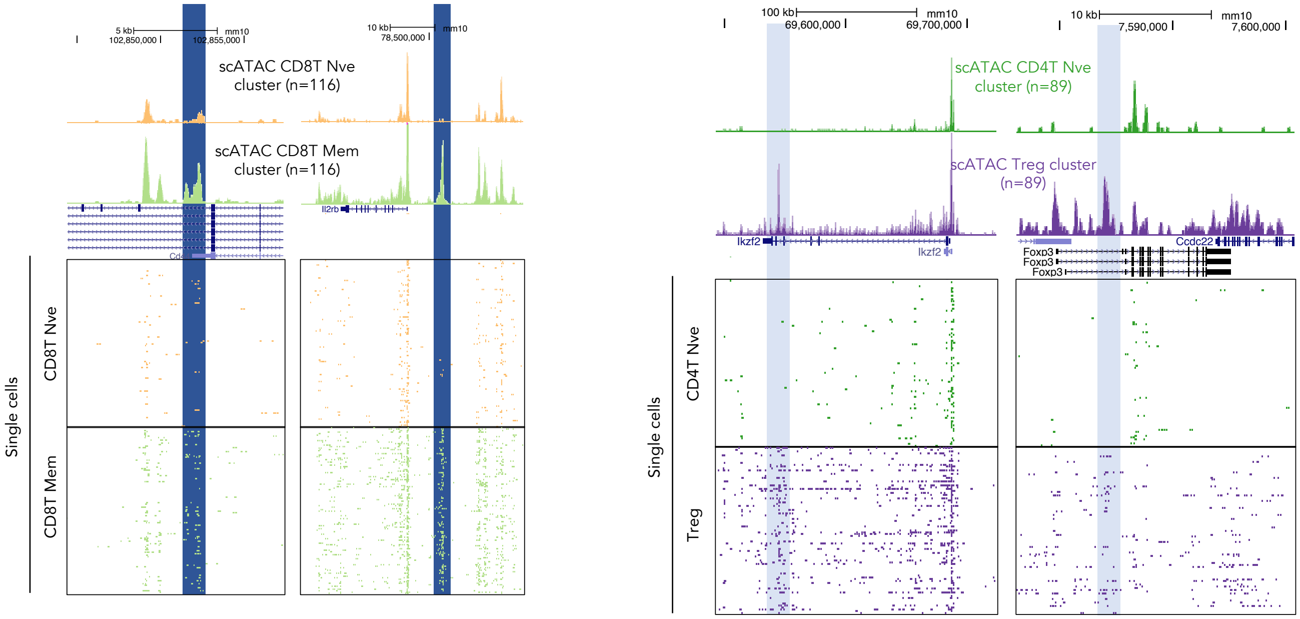

c

ImmGen NK cell RNA-seq sample	*Cd83* expression (tpm)
DC.4+.Sp#1	498.9
DC.4+.Sp#2	712.3
DC.4+.Sp#3	1007.9
NK.27-11b+.Sp#2	0
NK.27+11b+.Sp#1	0
NK.27+11b+.Sp#2	3.5

Supplementary Figure 10. (a and b) UCSC genome browser tracks showing ATAC-seq profiles of aggregate (top panel) and individual single cells (bottom panels). Known enhancers are highlighted. **(c)** *Cd83* expression from the ImmGen bulk RNA-seq of the indicated sample.

	cluster0	cluster1	cluster2	cluster3	cluster4	cluster5	cluster6	cluster7	cluster8	cluster9	cluster10	cluster11
**0**	Cd9	Fcer2a	Ptpn12	Myo10	Cdh26	Mafb	Cd8b1	Plxnd1	Lim2	Zdhhc2	Slc46a2	Ermard
**1**	Lyst	Chil4	Katnb1	Pdlim4	Dtx1	Mgat5b	A630075F10Rik	Psapl1	Kcng3	Nf2	Myo1g	Pofut2
**2**	Cxcr4	B3gnt7	Ypel1	Trub1	Ackr3	Smad3	5730508B09Rik	Mtx3	Vmn1r234	Spry1	Ctnna1	Magi2
**3**	Cd300a	Mir7211	S100z	Klhl5	Cd9	Eml6	Cd8a	Akap12	Mir3075	Mir21a	Dusp22	Rftn1
**4**	Cxcr2	Zfp407	Pigv	Itsn2	AW011738	Mad1l1	Kcnc1	Wnt8b	Gzma	Mpp2	Abcg3	Ccl3
**5**	Taldo1	Bmp2k	Mir7683	Polr1a	Taok3	Zfyve9	Cd8a	Myl10	9330199G10Rik	Ptpn13	Kit	Iqgap1
**6**	Map2k3	Xylt1	Gm6981	1700042G15Rik	Smyd2	Parp2	Nkg7	Olfr521	Smarcd3	Tank	Dpp4	Nol4l
**7**	Cxcl5	Wwox	Il2rb	Siah3	Mirlet7a-1	Hsf2	Cd8b1	Cplx2	Ccl3	Maf	Arhgap26	Gm7550
**8**	Nfe2	Stpg2	Mylip	Gm11186	Cdh26	Pdgfrb	Itgae	Thoc3	A730036I17Rik	Nr2e3	Macc1	Mir697
**9**	Tacstd2	1110059E24Rik	Gm13547	1700056E22Rik	Ackr3	Mtl5	Smad7	Thbs4	Lingo1	Tnfsf11	Abi1	Tpp2
**10**	Fam63a	Tmem252	Runx2	Cd4	Serpine2	Spred1	Haao	Kcnj1	1700063O14Rik	A630076J17Rik	Lonrf2	Frmd4b
**11**	Cxcr2	Rps15a	Tmevpg1	Gcm2	Thada	Gsdmd	Mir3108	Tnrc18	Snx20	Kbtbd11	Defb25	Tmem171
**12**	Rplp0	Tmprss13	Cdc42ep1	Gm13986	Dennd2d	4833417C18Rik	Tmed1	Nol4l	Cacna1c	Ctla4	A530013C23Rik	Klhl18
**13**	Tgif2	Zfp318	Ppp4r2	Epas1	Rgmb	Gm7168	Gosr1	Slc22a12	Bbc3	Acot11	H2-Aa	Bcl11b
**14**	Taldo1	AI427809	Gpr183	Itgb3	Dtx1	Atg4c	Ric8b	Cep89	Sept5	Axin2	Bloc1s2	Map3k8
**15**	Pygl	1700016K19Rik	Igsf23	Gm15417	LOC215458	Atp1b3	Orai2	Igf2bp3	Gpx8	B930018H19Rik	Npc1	Lrrc1
**16**	E2f2	Ciita	Dnaja2	Gcc1	Echdc3	F10	Efcc1	Nt5dc3	Zeb2	Art2b	Tomm20	Mir199a-1
**17**	Mir7021	Sorl1	Rgs3	Cdhr3	Mzb1	Ak2	2210416O15Rik	Wnt10a	Chpt1	Art2a-ps	Sh3bp4	4921513I03Rik
**18**	Slc2a3	Shank1	Sycp3	Olfr1510	Cdc42bpb	5430437J10Rik	Hmga2	Cpm	Gm29811	Asxl2	Jak2	Tnfrsf8
**19**	Ptma	Sacm1l	Gm38403	Gm13582	Gns	4930481A15Rik	Agpat4	Rrm2b	Pisd-ps1	Rgs16	Tbc1d8	Pold2
**20**	Tacstd2	H2-Aa	Abhd2	St8sia6	Myof	Pld1	Ccdc102a	Zfp608	Fcgr2b	Grb7	Gm13498	Fam131a
**21**	Fbxo31	Icosl	Cdk5rap3	Adar	Gpr55	AI463170	Cd8a	Cplx2	Chsy1	Rgs1	Jag1	Rasgrp1
**22**	Cxcr2	Osbpl10	Tmevpg1	Pdlim4	NaN	1700012I11Rik	Slc6a19	1700065J11Rik	Ptpre	Plxnc1	B230217C12Rik	Wisp2
**23**	Ifitm5	Sergef	Samd3	Mir151	4930581F22Rik	4930552P12Rik	Cd8a	Fads1	1700094M24Rik	Tjp2	Rnf216	Il20rb
**24**	Ckap4	Nup133	Smndc1	Sugct	Ptpn14	Plcb1	Mta3	Nfatc2	Pla1a	Ctla4	Ffar2	Emb
**25**	Zbtb16	Stap1	Abhd2	Zfp800	Cxcr4	2900026A02Rik	Hdac7	Igf2bp3	Scrg1	Eea1	Gm4814	Fbxo28
**26**	Tigd4	Pakap	Galr1	Cd200r3	Ubl3	Atp2b1	Runx3	Adcy9	Pik3r1	Pabpc2	Depdc1b	Gpr21
**27**	C130050O18Rik	H2-Aa	Drc1	Calcrl	Zfp36l1	Sowahc	Trpm1	Cecr2	Cmklr1	Fam76b	Flt3	Gpr25
**28**	Csf3r	Gpr137b-ps	Gabrp	Fam105a	Coro2b	Dagla	Nkg7	Man1c1	Gm2176	Lrig1	4833427F10Rik	Chd1
**29**	Rabac1	H2-Ea-ps	Dynlrb2	Dapk1	Setbp1	A230028O05Rik	Cd226	Gm38404	4930556N09Rik	Khdc1a	Zbtb46	Sacm1l
**30**	Nlrp12	Ppdpf	Kdm4d	4933406K04Rik	Blk	Pparg	Cd8b1	Bhlhe41	B230217C12Rik	Msl3l2	Acvr2a	Gsap
**31**	Emb	Cenpe	Txk	Tgfbr3	Mir1941	Nfkbiz	Ldlrad1	4930515G16Rik	Cox4i2	Mamstr	Mir1231	Fxyd3
**32**	Myh9	Icosl	Agtr1b	Slc19a3	Dtx1	Vcan	Gm29687	4930515G16Rik	Hgsnat	Nek7	Ddr1	Gpr25
**33**	Pgd	Enpp6	Cmc1	Myo10	Akap5	Gm4262	Col6a1	Cplx2	Pisd-ps3	Zfp41	Myo1h	Gdf1
**34**	Zeb2	Pxdc1	Mir7057	B4galt5	Sirpa	Xirp1	Wipf2	9430020K01Rik	Fastkd1	Tbc1d4	Zfp800	A630001G21Rik
**35**	Gcnt2	Capzb	Fosl2	Pno1	Prkcz	Ccr3	Cd8b1	Acaca	1700018C11Rik	Myo3b	Itpr1	Hemgn
**36**	Itgb2	H2-Eb1	Parp8	Epas1	Gm15713	Pola2	Cxcr4	Ldoc1l	Jam3	Gm10560	Serpina4-ps1	Gm5547
**37**	Susd1	Stoml1	Serpina12	Msra	Cmah	Galnt9	Irgc1	Tbc1d7	Kcnk13	Comt	Kif16b	Immp2l
**38**	Grina	Rgs9	Olfr525	Unc80	Kmt2a	Cmklr1	Slc6a19os	Bcl2l1	Efhd2	Ndfip1	Ccnd1	Gm7443
**39**	Mrpl33	Scd1	Gimap4	Ibtk	Hmgn3	Pld4	Trnp1	Gpr25	Adamts14	Gpr15	Gm9733	Nt5dc3
**40**	Dmxl2	Ptpn11	Fyn	Chl1	Atxn1	Msr1	Egfl7	Pik3c2b	Irf8	Suco	Grk3	Bcl2l10
**41**	Reep3	B3gat2	Btbd11	Chdh	Tsga13	Gsap	Cd8a	Gm12159	Id2	Lrrc32	Mefv	Rftn1
**42**	Cxcr1	Ezr	Runx2	Mtx2	Plac8	Commd9	Mir467h	2310061N02Rik	Nup50	Gm11985	Reps1	P2rx3
**43**	Slc11a1	Rasgef1b	Ccl5	Tnni1	Atxn1	4933433H22Rik	Ss18	Paqr6	Rps4l	Fxr1	Rrad	Rpl38
**44**	Slmap	9030404E10Rik	Gm38403	Cck	Syk	Arhgef10l	Sos1	Guca2b	Sytl2	Aven	Rgs2	Mir7235
**45**	Csf3r	Helz2	Nosip	Zscan10	Cbx4	Pitpna	Ccdc102a	Dcun1d1	St5	1700016G22Rik	Frmd5	Phf3
**46**	Arntl2	Chrna9	Runx2	Fam65b	Fasl	Tm9sf4	Il21r	Prkcg	Znrf2	Ptger2	Cdyl2	Gm7008
**47**	Ndel1	Parp8	Kcnj8	Cers6	Rps24	Selenbp1	Bfsp2	Gm17455	Nav2	Slc25a19	Tbc1d4	Serpina3f
**48**	Ptafr	March1	Mapre2	Kif23	Slc31a2	Zfp397	Cst7	Gopc	Clnk	Ets1	Mreg	Manba
**49**	Map7	Akap13	Fam169b	Rab11fip4	Dusp16	Agap1	Ap1ar	Myb	Gm20750	Sdcbp2	BC039771	Erc2

Supplementary Figure 11. Nearest genes assigned to the top 50 marker peaks in each single cell cluster.

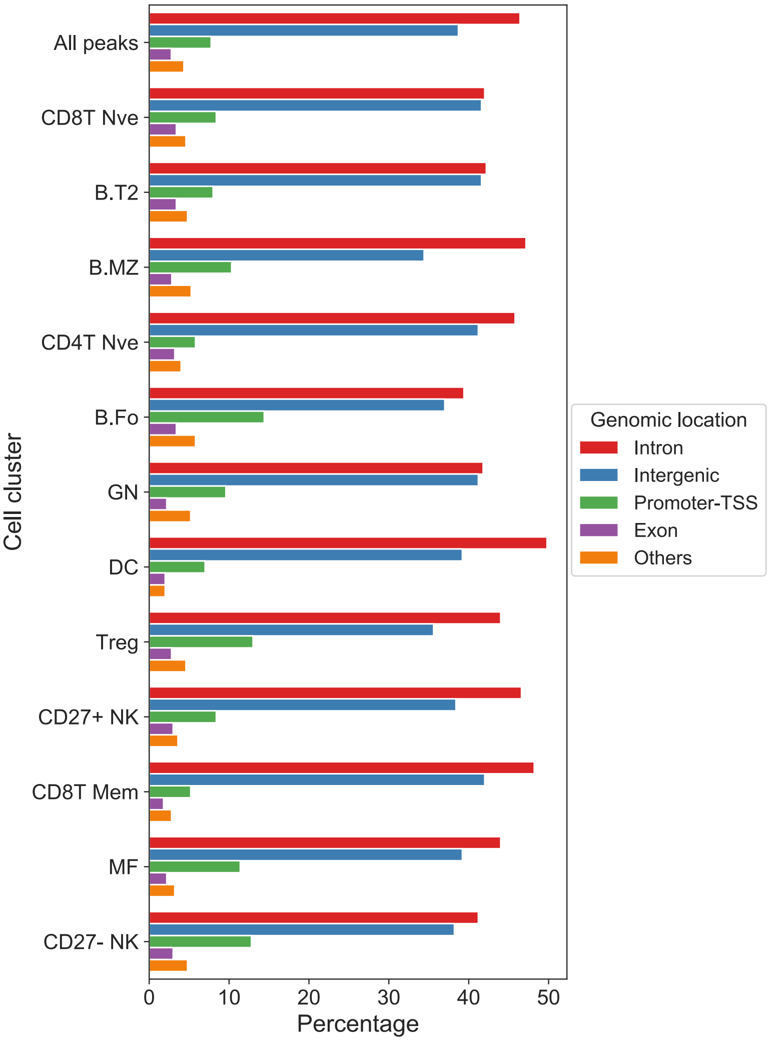

Supplementary Figure 12. Genomic distribution (by HOMER) of all peaks and the marker peaks in each single cell cluster.

Supplementary Methods

Protocol for plate-based scATAC-seq using FACS
Timestamp: 15-Feb-2018

1. One day before the experiment, prepare the plates by aliquoting 2 µl 2X Lysis Buffer to each well of the plates (either 96-well or 384-well plate). Then add 2 µl of 10 µM S5xx/N7xx Nextera Index Primer Mix (5 µM each) to each well. Seal the plate and store in -80 °C.

   Recipe for 2X Lysis Buffer:

100 mM	Tris.HCl, pH 8.0
100 mM	NaCl
40 µg/ml	Proteinase K (Ambion, AM2546, 20 mg/ml stock)
0.4%	SDS

2. On the day of the experiment, thaw plates at room temperature.
3. Pre-coat all tubes with 500 µl 0.5% BSA (prepared in 1X PBS) for a few minutes to reduce sample loss. Count or sort 5k - 50k cells into 1.5-ml eppendorf tubes. DO NOT use DNA LoBind tubes for pelleting cells, which does not work well especially when cell numbers are limited.
4. Pellet cells at 500 g, 4 °C, 5 minutes.
5. Wash the cell pellet with 100 µl ice-cold PBS, twice, 500 g, 4 °C, 5 minutes, and carefully remove the supernatant.
6. Resuspend the cell pellet in 50 µl tagmentation mix. The recipe for the tagmentation mix is as follows (THS-seq recipe):

12.5 µl	4X THS-seq TD buffer
5 µl	10X Digitonin
27. 5 µl	H_2O
5 µl	Illumina Tn5 (Nextera kit, Illumina Cat No. FC-121-1030)

Recipe for 4X THS-seq TD buffer:

132 mM	Tris-acetate, pH 7.8
264 mM	Potassium acetate
40 mM	Magnesium acetate
64%	Dimethylformamide (DMF)

Recipe for 10X Digitonin:

1 µl	Digitonin (Promega, G9441, 2% stock)
19 µl	H_2O

7. Put the tagmentation reaction (50 µl) on a thermomixer, 37 °C, 800 rpm, 30 minutes.
8. Stop the reaction by adding 50 µl tagmentation stop buffer (TSB). Recipe for TSB:

10 mM	Tris-HCl, pH 8.0
20 mM	EDTA, pH 8.0

9. Leave on ice for 10 minutes.
10. Add 100 - 300 µl PBS/0.5% BSA to the 100 µl stopped tagmentation mix, and transfer to a FACS tube.
11. Optional: add DAPI to stain nuclei based on manufacturer's instruction.
12. Sort DAPI positive single nuclei into the plates prepared the day before.
13. Quickly spin down and seal the plate well (can be stored in -80 °C for a few weeks from here), and put the plate on a PCR machine, with lid temperature set to 100 °C.
14. Incubate the plate at 65 °C for 15 minutes to perform Tn5 release.
15. Add equal volume (4 µl) of 10% TWEEN-20 to each well to quench SDS. Briefly vortex to mix.
16. Add 2 µl H_2O to each well.
17. Add 10 µl 2X NEBNext® High-Fidelity 2X PCR Master Mix (NEB M0541L) to each well

18. At this stage, each well contains 20 µl PCR reaction.
19. Perform library amplification PCR:

    72 °C 10 minutes

    98 °C 5 minutes

    [98 °C 10 seconds, 63 °C 30 seconds, 72 °C 20 seconds] x 18

    10 °C hold
20. Combine all reactions into a 50-ml falcon, which yields about 20 µl x 384 = 7.68 ml. Normally, the yield will be ~ 7.2 ml.
21. Add 5 volumes (~ 36 ml) Buffer PB (Qiagen), mix well, and pass reaction volume through a single column from a Qiagen MinElute PCR Purification Kit by connecting the column to a vacuum.
22. To wash the column, pass through 40 ml Column Wash Buffer (10 mM Tris-HCl, pH 7.5, 80% ethanol).
23. Spin down the column at top speed on a table top centrifuge to remove all traces of ethanol, and remember to use a pipette to remove the ethanol leftover on the rim of the Qiagen column.
24. Elute the library in 12.5 µl Buffer EB. Perform the elution three times and combine the three elutes to a final volume of ~ 36 µl.
25. Do a final fragment size selection using 0.5X SPRI upper cutoff, followed by 1.2X SPRI lower cutoff, and elute in 30 µl 10 mM Tris-HCl, pH 8.0.
26. Run Nanodrop to obtain a rough estimate of the concentration, and then dilute the library to a range suitable for Bioanalyzer/TapeStation etc.
27. Check for expected results (see Supplementary Fig. 2a).
28. Sequencing: we sequenced each 384 pool on one lane of Hiseq 2000 or one rapid run of Hiseq 2500, which nearly saturated the library. From the data obtained, each cell was sequenced to about 1 million reads, but only ~30,000 unique reads were obtained per cell. Further reads were redundant, which is comparable (if not better) to published scATAC-seq by other methods. Theoretically, 30,000 reads per cell should be sufficient to profile the unique reads. However, considering the presence of mitochondrial DNA, non-mapped and non-uniquely mapped reads, it is safer to aim for at least 100,000 reads per cell.

Oligonucleotides sequence

N701 CAAGCAGAAGACGGCATACGAGATTCGCCTTAGTCTCGTGGGCTCGG

N702 CAAGCAGAAGACGGCATACGAGATCTAGTACGGTCTCGTGGGCTCGG

N703 CAAGCAGAAGACGGCATACGAGATTTCTGCCTGTCTCGTGGGCTCGG

N704 CAAGCAGAAGACGGCATACGAGATGCTCAGGAGTCTCGTGGGCTCGG

N705 CAAGCAGAAGACGGCATACGAGATAGGAGTCCGTCTCGTGGGCTCGG

N706 CAAGCAGAAGACGGCATACGAGATCATGCCTAGTCTCGTGGGCTCGG

N707 CAAGCAGAAGACGGCATACGAGATGTAGAGAGGTCTCGTGGGCTCGG

N710 CAAGCAGAAGACGGCATACGAGATCAGCCTCGGTCTCGTGGGCTCGG

N711 CAAGCAGAAGACGGCATACGAGATTGCCTCTTGTCTCGTGGGCTCGG

N712 CAAGCAGAAGACGGCATACGAGATTCCTCTACGTCTCGTGGGCTCGG

N714 CAAGCAGAAGACGGCATACGAGATTCATGAGCGTCTCGTGGGCTCGG

N715 CAAGCAGAAGACGGCATACGAGATCCTGAGATGTCTCGTGGGCTCGG

N716 CAAGCAGAAGACGGCATACGAGATTAGCGAGTGTCTCGTGGGCTCGG

N718 CAAGCAGAAGACGGCATACGAGATGTAGCTCCGTCTCGTGGGCTCGG

N719 CAAGCAGAAGACGGCATACGAGATTACTACGCGTCTCGTGGGCTCGG

N720 CAAGCAGAAGACGGCATACGAGATAGGCTCCGGTCTCGTGGGCTCGG

N721 CAAGCAGAAGACGGCATACGAGATGCAGCGTAGTCTCGTGGGCTCGG

N722 CAAGCAGAAGACGGCATACGAGATCTGCGCATGTCTCGTGGGCTCGG

N723 CAAGCAGAAGACGGCATACGAGATGAGCGCTAGTCTCGTGGGCTCGG

N724 CAAGCAGAAGACGGCATACGAGATCGCTCAGTGTCTCGTGGGCTCGG

N726 CAAGCAGAAGACGGCATACGAGATGTCTTAGGGTCTCGTGGGCTCGG

N727 CAAGCAGAAGACGGCATACGAGATACTGATCGGTCTCGTGGGCTCGG

N728 CAAGCAGAAGACGGCATACGAGATTAGCTGCAGTCTCGTGGGCTCGG

N729 CAAGCAGAAGACGGCATACGAGATGACGTCGAGTCTCGTGGGCTCGG

S502 AATGATACGGCGACCACCGAGATCTACACCTCTCTATTCGTCGGCAGCGTC

S503 AATGATACGGCGACCACCGAGATCTACACTATCCTCTTCGTCGGCAGCGTC

S505 AATGATACGGCGACCACCGAGATCTACACGTAAGGAGTCGTCGGCAGCGTC

S506 AATGATACGGCGACCACCGAGATCTACACACTGCATATCGTCGGCAGCGTC

S507 AATGATACGGCGACCACCGAGATCTACACAAGGAGTATCGTCGGCAGCGTC

S508 AATGATACGGCGACCACCGAGATCTACACCTAAGCCTTCGTCGGCAGCGTC

S510 AATGATACGGCGACCACCGAGATCTACACCGTCTAATTCGTCGGCAGCGTC

S511 AATGATACGGCGACCACCGAGATCTACACTCTCTCCGTCGTCGGCAGCGTC

S513 AATGATACGGCGACCACCGAGATCTACACTCGACTAGTCGTCGGCAGCGTC

S515 AATGATACGGCGACCACCGAGATCTACACTTCTAGCTTCGTCGGCAGCGTC

S516 AATGATACGGCGACCACCGAGATCTACACCCTAGAGTTCGTCGGCAGCGTC

S517 AATGATACGGCGACCACCGAGATCTACACGCGTAAGATCGTCGGCAGCGTC

S518 AATGATACGGCGACCACCGAGATCTACACCTATTAAGTCGTCGGCAGCGTC

S520 AATGATACGGCGACCACCGAGATCTACACAAGGCTATTCGTCGGCAGCGTC

S521 AATGATACGGCGACCACCGAGATCTACACGAGCCTTATCGTCGGCAGCGTC

S522 AATGATACGGCGACCACCGAGATCTACACTTATGCGATCGTCGGCAGCGTC
