# Supplementary material for: A rapid and robust method for single cell chromatin accessibility profiling
Source: Nat Commun. 2018 Dec 17;9:5345. doi: 10.1038/s41467-018-07771-0 (PMC6297232; doi:10.1038/s41467-018-07771-0)
Supplement: Supplementary file 7 — Description of Additional Supplementary Files [file 41467_2018_7771_MOESM7_ESM.docx]

**Title:** Supplementary Data 1:
**Description:** The detailed summary of different quality control metrics on all cells tested in this study.

**Title:** Supplementary Data 2:
**Description:** The detailed annotation of each mouse splenocyte, including the quality control metrics and predicted cell types.

**Title:** Supplementary Data 3:
**Description:** Annotation of the top 500 marker peaks in each cell cluster.

**Title:** Supplementary Data 4:
**Description:** HOMER known motif enrichment results of the top 500 marker peaks in each cell cluster.
