# Supplementary material for: A rapid and robust method for single cell chromatin accessibility profiling
Source: Nat Commun. 2018 Dec 17;9:5345. doi: 10.1038/s41467-018-07771-0 (PMC6297232; doi:10.1038/s41467-018-07771-0)
Supplement: Supplementary file 8 — Reporting Summary [file 41467_2018_7771_MOESM8_ESM.pdf]

nature research

Corresponding author(s): Sarah Teichmann

Reporting Summary

Nature Research wishes to improve the reproducibility of the work that we publish. This form provides structure for consistency and transparency in reporting. For further information on Nature Research policies, see Authors & Referees and the Editorial Policy Checklist.

Statistical parameters

When statistical analyses are reported, confirm that the following items are present in the relevant location (e.g. figure legend, table legend, main text, or Methods section).

n/a	Confirmed	
☐	☒	The exact sample size (n) for each experimental group/condition, given as a discrete number and unit of measurement
☐	☒	An indication of whether measurements were taken from distinct samples or whether the same sample was measured repeatedly
☐	☒	The statistical test(s) used AND whether they are one- or two-sided<br>*Only common tests should be described solely by name; describe more complex techniques in the Methods section.*
☐	☒	A description of all covariates tested
☐	☒	A description of any assumptions or corrections, such as tests of normality and adjustment for multiple comparisons
☐	☒	A full description of the statistics including central tendency (e.g. means) or other basic estimates (e.g. regression coefficient) AND variation (e.g. standard deviation) or associated estimates of uncertainty (e.g. confidence intervals)
☐	☒	For null hypothesis testing, the test statistic (e.g. F, t, r) with confidence intervals, effect sizes, degrees of freedom and P value noted<br>*Give P values as exact values whenever suitable.*
☒	☐	For Bayesian analysis, information on the choice of priors and Markov chain Monte Carlo settings
☒	☐	For hierarchical and complex designs, identification of the appropriate level for tests and full reporting of outcomes
☐	☒	Estimates of effect sizes (e.g. Cohen's d, Pearson's r), indicating how they were calculated
☒	☐	Clearly defined error bars<br>*State explicitly what error bars represent (e.g. SD, SE, CI)*

Our web collection on statistics for biologists may be useful.

Software and code

Policy information about availability of computer code

Data collection	iRODS Version 4.2.2
Data analysis	bedtools v2.27.1, cutadapt v1.16, hisat2 v2.1.0, homer v4.9.1, matplotlib v2.2.2, numpy v1.14.2, pandas v0.22.0, picard v2.17.10, salmon v0.9.1, samtools v1.7, scikit-learn v0.19.1, scipy v1.0.1, seaborn v0.8.1, seqtk v1.2, snakemake v4.7.0. All codes are deposited at the GitHub repository: https://github.com/dbrg77/plate_scATAC-seq

For manuscripts utilizing custom algorithms or software that are central to the research but not yet described in published literature, software must be made available to editors/reviewers upon request. We strongly encourage code deposition in a community repository (e.g. GitHub). See the Nature Research guidelines for submitting code & software for further information.

Data

Policy information about availability of data

All manuscripts must include a data availability statement. This statement should provide the following information, where applicable:

- Accession codes, unique identifiers, or web links for publicly available datasets
- A list of figures that have associated raw data
- A description of any restrictions on data availability

The sequencing data has been deposited at ArrayExpress, accession number E-MTAB-6714 [https://www.ebi.ac.uk/arrayexpress/experiments/E-MTAB-6714/]. The

code used for the analysis is available on the Github repository https://github.com/dbrg77/plate_scATAC-seq. The UCSC genome browser tracks containing both the ImmGen bulk ATAC-seq and scATAC-seq from this study can be viewed via this link: http://genome-euro.ucsc.edu/cgi-bin/hgTracks?hgS_doOtherUser=submit&hgS_otherUserName=dbrg77&hgS_otherUserSessionName=mSpleen_scATAC_cluster.

Field-specific reporting

Please select the best fit for your research. If you are not sure, read the appropriate sections before making your selection.

☒ Life sciences ☐ Behavioural & social sciences ☐ Ecological, evolutionary & environmental sciences

For a reference copy of the document with all sections, see nature.com/authors/policies/ReportingSummary-flat.pdf

Life sciences study design

All studies must disclose on these points even when the disclosure is negative.

Sample size	Since only a limited number of scATAC-seq studies are published, it is difficult to determine the sample size (cell number) in advance. However, for the purpose of simply assessing whether a scATAC-seq experiment works or not, 96 cells are generally enough. We have profiled more than 3,000 cells in our studies.
Data exclusions	For the deposition of the raw data, there is no exclusion, all single cells, including failed ones, are deposited into ArrayExpress. For data analysis, we exclude cells that failed our QC (too few reads and low mapping rate) and noisy/low-coverage cells (bottom 5% in terms of number of peaks detected in the single cell).
Replication	Two spleens were profiled from two mice in different days. In total, 3,648 splenocytes were profiled, and the smallest cell cluster identified have 55 cells.
Randomization	Single nuclei were randomly sorted into 384-well plates by FACS.
Blinding	N/A

Reporting for specific materials, systems and methods

Materials & experimental systems

n/a	Involved in the study
☒	☐ Unique biological materials
☐	☒ Antibodies
☐	☒ Eukaryotic cell lines
☒	☐ Palaeontology
☐	☒ Animals and other organisms
☒	☐ Human research participants

Methods

n/a	Involved in the study
☒	☐ ChIP-seq
☐	☒ Flow cytometry
☒	☐ MRI-based neuroimaging

Antibodies

Antibodies used	anti-Mouse CD4 PE (eBioscience cat no. 12-0043-82)
Validation	Validated by the manufacturer by FACS.

Eukaryotic cell lines

Policy information about cell lines

Cell line source(s)	K-562 (ATCC® CCL-243™)<br>E14 mESCs were obtained from Pentao Liu's lab.<br>293T (ATCC® CRL-3216™)<br>NIH/3T3 (ATCC® CRL-1658™)
Authentication	K562, 293T and NIH/3T3 authenticated by ATCC, and E14 mESCs were authenticated by a previous study (Nature volume 550, 393–397)

Mycoplasma contamination	Tested negative.
Commonly misidentified lines (See ICLAC register)	No commonly misidentified cell lines were used.

Animals and other organisms

Policy information about studies involving animals; ARRIVE guidelines recommended for reporting animal research

Laboratory animals	Adult male C57BL/6Jax mice.
Wild animals	None.
Field-collected samples	None

Flow Cytometry

Plots

Confirm that:

☒ The axis labels state the marker and fluorochrome used (e.g. CD4-FITC).

☒ The axis scales are clearly visible. Include numbers along axes only for bottom left plot of group (a 'group' is an analysis of identical markers).

☒ All plots are contour plots with outliers or pseudocolor plots.

☒ A numerical value for number of cells or percentage (with statistics) is provided.

Methodology

Sample preparation	The spleen from a C57BL/6Jax mouse was mashed by a 2-ml syringe plunger through a 70 µm cell strainer into 30 ml 1X DPBS supplied with 2 mM EDTA and 0.5% (w/v) BSA. Cells were centrifuged down, supernatant was removed, and the cell pellet was briefly vortexed. 5 ml 1X RBC lysis buffer was used to resuspend the cell pellet, and the cell suspension was vortexed again, and left on bench for 5 minutes to lyse red blood cells. Then 45 ml 1X DPBS was added, and cells were centrifuged down. 30 ml 1X DPBS were used to resuspend the cell pellet. The cell suspension was passed through a Miltenyi 30 µm Pre-Separation Filter, and the cell number was determined using C-chip counting chamber. All centrifugations were done at 500 g, 4 °C, 5 minutes.
Instrument	BD INFLUX (Becton Dickinson UK Ltd)
Software	FACS is merely used as a single cell isolation tool. No further analysis is done.
Cell population abundance	N/A.
Gating strategy	N/A (all DAPI positive).

☒ Tick this box to confirm that a figure exemplifying the gating strategy is provided in the Supplementary Information.
