# Supplementary material for: A rapid and robust method for single cell chromatin accessibility profiling
Source: Nat Commun. 2018 Dec 17;9:5345. doi: 10.1038/s41467-018-07771-0 (PMC6297232; doi:10.1038/s41467-018-07771-0)
Supplement: Supplementary file 2 — Peer Review File [file 41467_2018_7771_MOESM2_ESM.pdf]

This manuscript has been previously reviewed at another journal that is not operating a transparent peer review scheme. This document only contains reviewer comments and rebuttal letters for versions considered at Nature Communications. Mentions of the other journal have been redacted.

Reviewers' Comments:

Reviewer #1:

Remarks to the Author:

The authors have adequately addressed the specific/technical concerns raised by the reviewers. While the conceptual concern remains whether this method can enable research that would not be possible with other assays, I can follow the authors' argument, namely that a method that is a bit easier to implement than its alternatives may find its users even if neither the data quality nor the throughput is clearly better.

Response to reviewers’ comments.

Reviewer #1 (Remarks to the Author):

The authors have adequately addressed the specific/technical concerns raised by the reviewers. While the conceptual concern remains whether this method can enable research that would not be possible with other assays, I can follow the authors' argument, namely that a method that is a bit easier to implement than its alternatives may find its users even if neither the data quality nor the throughput is clearly better. We thank the reviewer for the comments.
